# Supplementary material for: Targeting BC200/miR218-5p Signaling Axis for Overcoming Temozolomide Resistance and Suppressing Glioma Stemness
Source: Cells. 2020 Aug 8;9(8):1859. doi: 10.3390/cells9081859 (PMC7463574; doi:10.3390/cells9081859)
Supplement: Supplementary file 1 [file cells-09-01859-s001.pdf]

SUPPLEMENTARY INFORMATION

Targeting BC200/miR218-5p signaling axis for overcoming temozolomide resistance and suppressing glioma stemness

Disclosed by Yu-Kai Su [1,2,3,4†], Jia Wei Lin[1,2,3,4†], Jing-Wen Shih[5,6], Hao-Yu Chuang[7], Iat-Hang Fong[2,3,4], Chi-Tai Yeh [1,8,9], Chien-Min Lin [1,2,3,4,*]

Affiliation:

1. Graduate Institute of Clinical Medicine, College of Medicine, Taipei Medical University, Taipei City 11031, Taiwan
2. Department of Neurology, School of Medicine, College of Medicine, Taipei Medical University, Taipei City 11031, Taiwan
3. Division of Neurosurgery, Department of Surgery, Taipei Medical University-Shuang Ho Hospital, New Taipei City 23561, Taiwan
4. Taipei Neuroscience Institute, Taipei Medical University, Taipei 11031, Taiwan
5. Graduate Institute of Cancer Biology and Drug Discovery, College of Medical Science and Technology, Taipei Medical University, Taipei 11031, Taiwan.
6. Ph.D. Program for Cancer Biology and Drug Discovery, College of Medical Science and Technology, Taipei Medical University, Taipei 11031, Taiwan.
7. Department of Neurosurgery, An Nan Hospital, China Medical University, Tainan 70965, Taiwan
8. Department of Medical Research & Education, Taipei Medical University-Shuang Ho Hospital, New Taipei City 23561, Taiwan
9. Department of Medical Laboratory Science and Biotechnology, Yuanpei University of Medical Technology, Hsinchu 300, Taiwan

† Contributed equally to this work

*Corresponding author

Dr. Chien-Min Lin, Division of Neurosurgery, Department of Surgery, Taipei Medical University-Shuang Ho Hospital, New Taipei City 23561, Taiwan. E-mail: m513092004@tmu.edu.tw

Supplementary Table S1.

No.	Target	Dilution	Source	
1	MRP1	1:200	ab24102	Abcam
2	Sox2	1:1000	ab137385	Abcam
3	MGMT	1:1000	#2739	cellsignaling
4	MLH1	1:1000	#3515	cellsignaling
5	MSH2	1:1000	#2017	cellsignaling
6	MSH6	1:1000	#5424	cellsignaling
7	N-Cadherin	1:1000	#13116	cellsignaling
8	Vimentin	1:1000	#5741	cellsignaling
9	Slug	1:1000	#9585	cellsignaling
10	Oct-4A	1:1000	#2890	cellsignaling
11	KLF4	1:1000	#12173	cellsignaling
12	Mut-P53	1:50	PAb240	Merck
13	β-actin	1:10000	66009-1-Ig	PROTEINTECH
14	BCRP1	1:1000	sc-58222	Santa Cruz
15	MDR1	1:1000	sc-55510	Santa Cruz
16	PMS2	1:1000	sc-25315	Santa Cruz
17	Ki-67	1:100	790-4286	Ventana

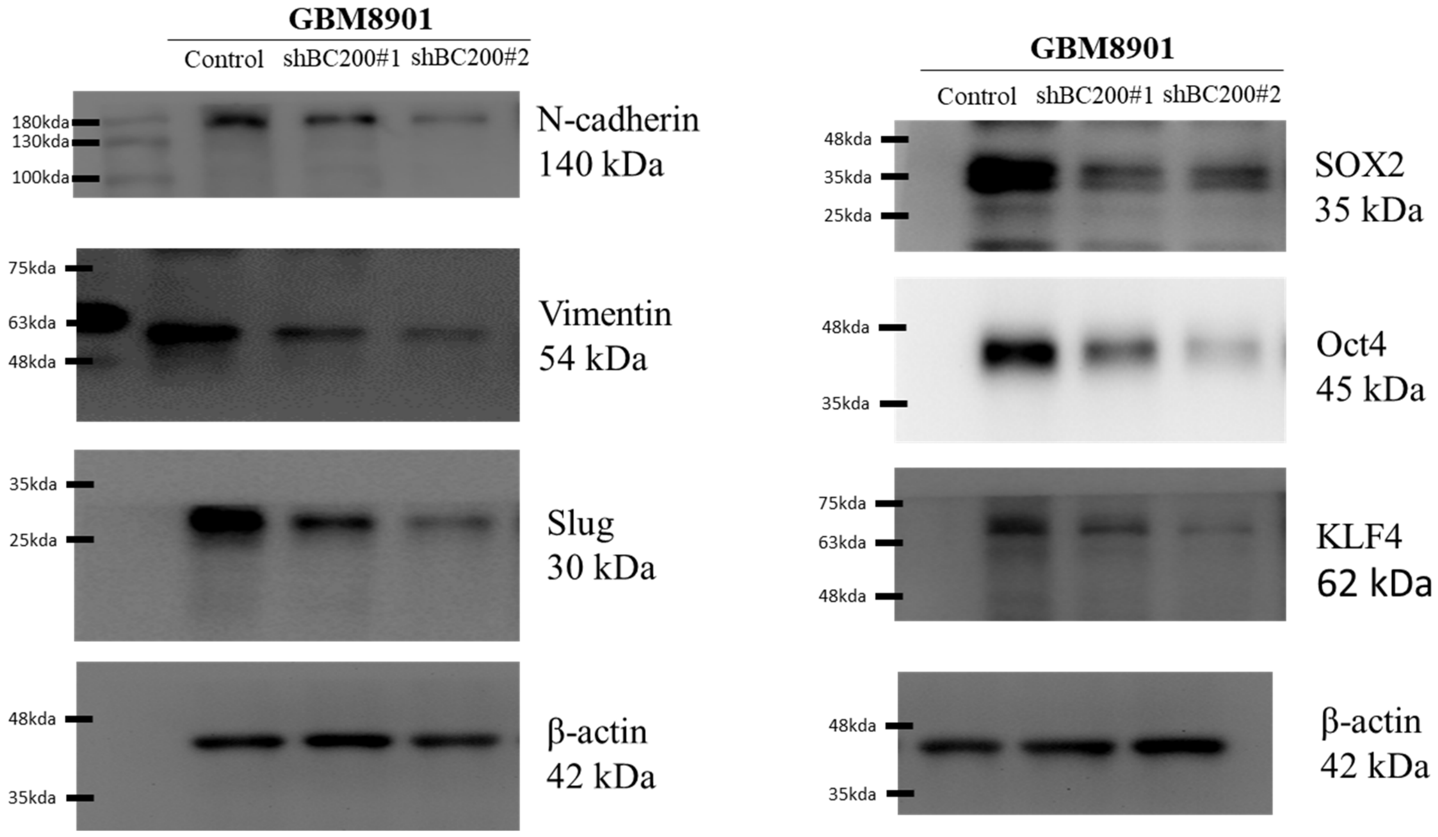

Supplementary Figure S1. Full-size blots of Figure 3D and 3H

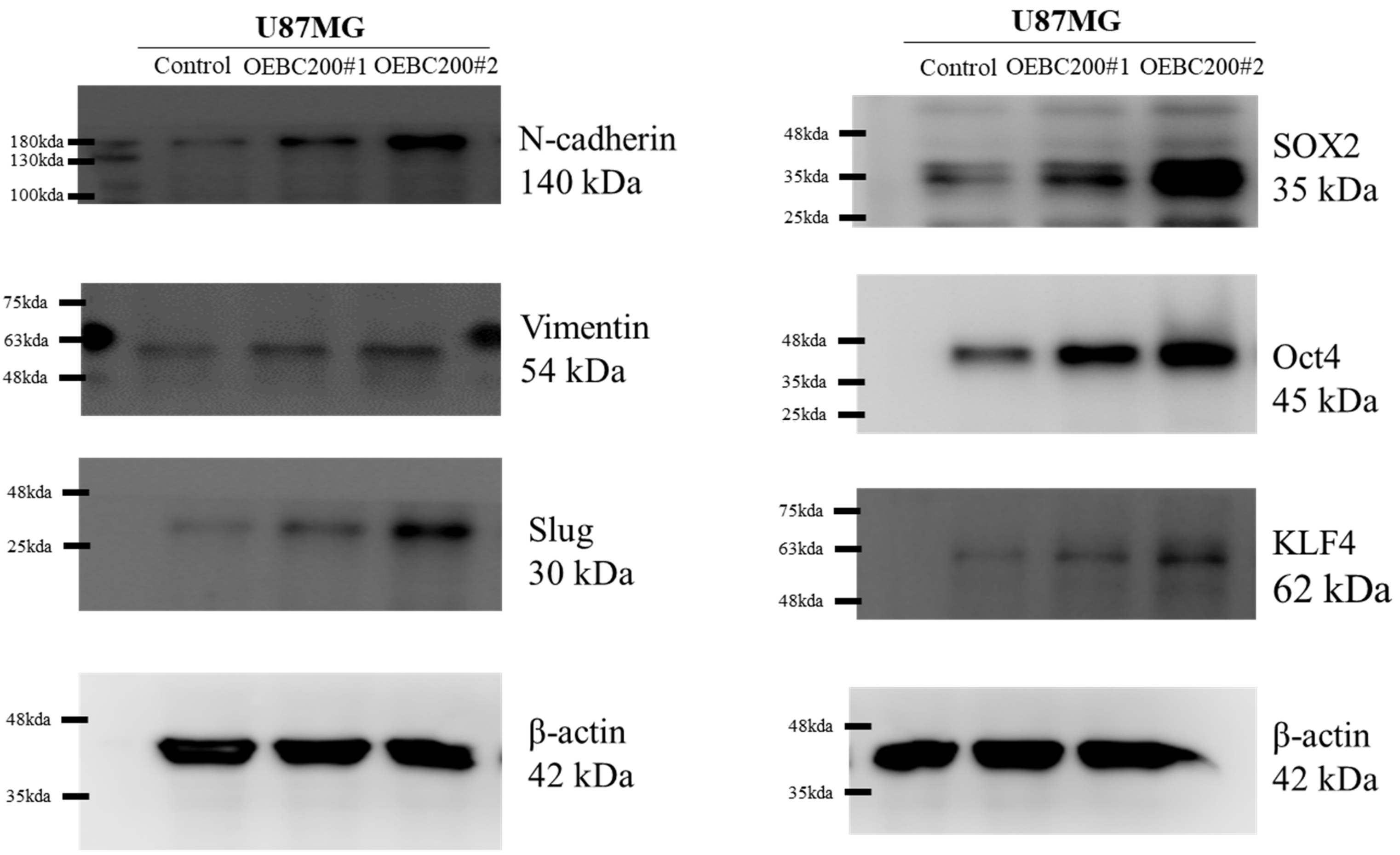

Supplementary Figure S2. Full-size blots of Figure 4D and 4H

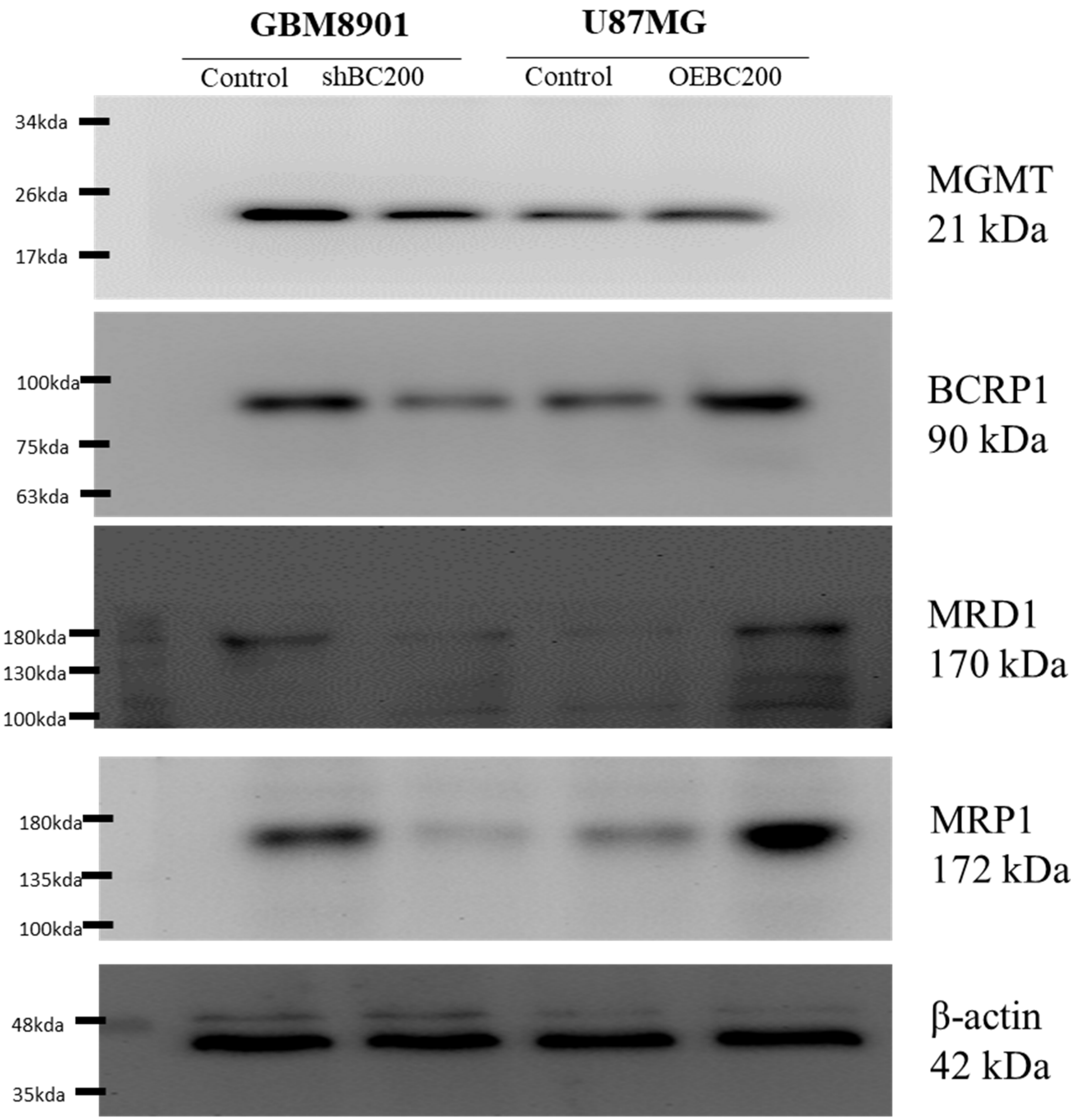

Supplementary Figure S3. Full-size blots of Figure 5B

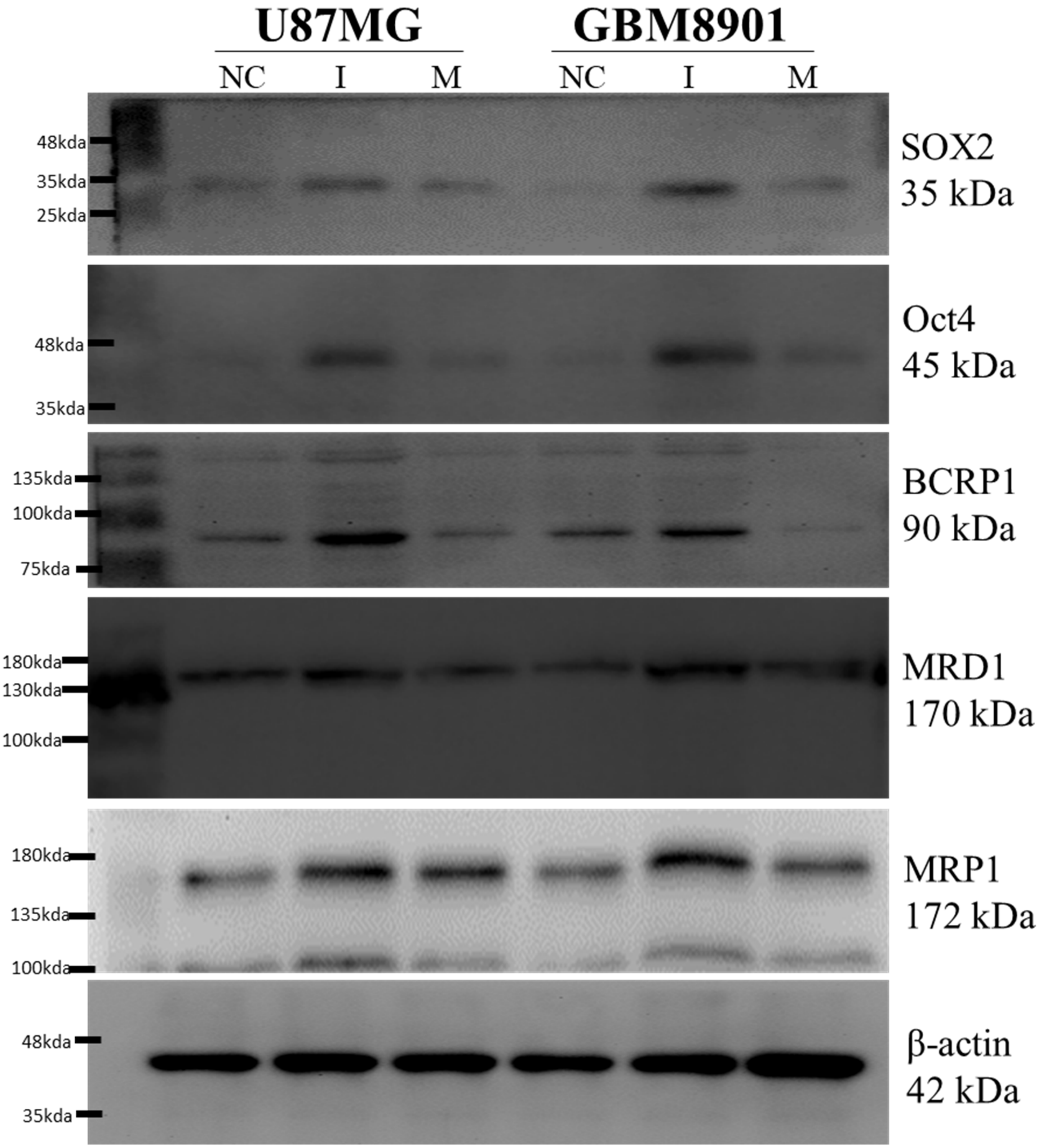

Supplementary Figure S4. Full-size blots of Figure 6G

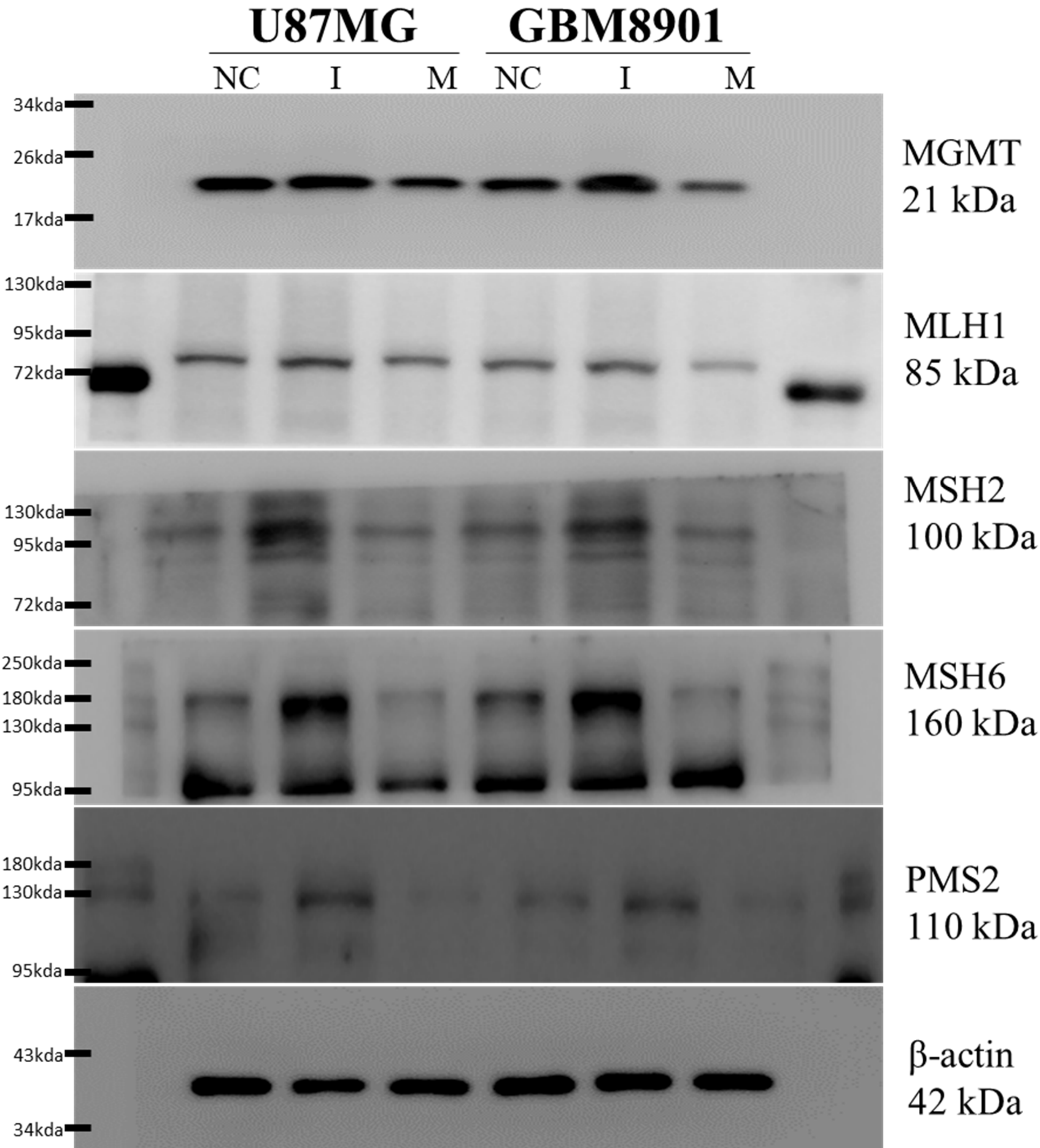

Supplementary Figure S5. Full-size blots of Figure 6H
